# Special Issue: Biomaterials for Dental and Orthopedic Applications

**DOI:** 10.3390/ijms27052122

**Published:** 2026-02-25

**Authors:** Lia Rimondini, Elena Varoni

**Affiliations:** 1 Department of Health Sciences, Università of Piemonte Orientale, 28100 Novara, Italy; 2 Department of Biomedical, Surgical and Dental Sciences, Università degli Studi di Milano, 20142 Milan, Italy; elena.varoni@unimi.it

Biomaterials play a pivotal role in contemporary dental and orthopedic applications, where tissue regeneration, functional restoration, and long-term clinical reliability remain major clinical challenges. By interacting dynamically with biological systems, biomaterials provide not only structural support but also biochemical and biophysical cues that regulate cellular behavior, tissue integration, and healing processes. For these reasons, biomaterials represent a cornerstone of regenerative medicine and advanced implantology.

Traditionally, metals and alloys, ceramics, polymers, composites, and nature-derived materials have constituted the backbone of dental and orthopedic therapies. However, increasing patient heterogeneity, complex pathological conditions, and higher expectations in terms of implant longevity and functionality have progressively exposed the limitations of standardized approaches. As a result, current research is increasingly focused on the personalized and precise application of biomaterials and medical devices, with the aim of tailoring material properties, surface characteristics, and biological performance to individual clinical needs.

This Special Issue was conceived to address this evolving paradigm by providing a multidisciplinary platform for innovative research on advanced biomaterials and enabling technologies for dental and orthopedic applications. The published contributions collectively illustrate how personalization can be achieved at multiple and interconnected levels, spanning material design, surface engineering, biological response assessment, computational modeling, and translational validation.

Several of the included studies focused on bioactive and nanostructured materials designed to actively promote tissue regeneration. In the dental field, gelatin-modified mesoporous bioactive glass demonstrated efficient blocking of dentinal tubules, enhanced deep mineralization, and improved biocompatibility, representing a promising strategy for dentin hypersensitivity treatment (contribution 1).

A substantial body of contributions addressed implant surface engineering, emphasizing its central role in modulating biological responses. Laser-produced microchannel structures on titanium surfaces significantly enhanced pre-osteoblast proliferation, osteogenic differentiation, and extracellular mineralization when compared with conventional surface treatments (contribution 2). Complementarily, computational fluid dynamics modeling demonstrated how surface microtopography and superhydrophilicity synergistically influence protein and blood recruitment at the implant interface, thereby modulating the early biological events that govern osseointegration (contribution 3).

The importance of condition-specific and patient-tailored biomaterials was demonstrated in in vivo studies focused on bone regeneration. Collagen-based scaffolds enriched with nanohydroxyapatite, with or without elastin, supported effective bone repair in both healthy and ovariectomized rat models, demonstrating osteoconductive behavior even under compromised metabolic conditions (contribution 4). These findings underscore the relevance of adapting biomaterial composition to systemic factors such as osteoporosis, which are increasingly prevalent in clinical practice.

At the cellular and subcellular levels, investigations into mitochondrial redox balance and oxidative stress responses of fibroblasts exposed to diversely anodized titanium alloys highlighted the complex relationship between surface treatments, ion release, oxidative stress, and apoptosis, reinforcing the need for continued optimization and the exploration of alternative or biodegradable materials (contribution 5).

Advanced analytical approaches were also represented in this Special Issue. A machine-learning-assisted proteomic analysis of extracellular vesicles derived from mesenchymal stem cells cultured on doped bioactive glass substrates enabled the detection of subtle, condition-dependent protein expression patterns not fully captured using classical statistical methods (contribution 6). The findings of this study underscore the growing importance of artificial intelligence and unconventional data analysis strategies in precision biomaterials research.

Finally, the authors of a comprehensive review focused on surface modification strategies for nickel–titanium (NiTi) shape memory alloys, with particular emphasis on low-temperature glow discharge plasma oxidation techniques, highlighted recent advancements aimed at enhancing corrosion resistance, reducing nickel ion release, and promoting osseointegration (contribution 7). The review authors also identified key challenges related to scalability, reproducibility, and clinical validation—critical aspects for successful translation.

Taken together, the contributions compiled in this first edition clearly delineate the scientific trajectory of the field. Bioactive and nanostructured materials for dentin and bone regeneration (contribution 1,4), precision surface engineering of metallic implants (contribution 2,3,6,7), and advanced biological and computational analyses (contribution 3,6) collectively illustrate that future progress in dental and orthopedic biomaterials will increasingly depend on the integration of personalized material design, advanced manufacturing technologies, biological precision, and clinical validation.

These converging research directions form the scientific foundation of the second edition of the Special Issue “Biomaterials for Dental and Orthopedic Applications”, which seeks to consolidate and expand these themes. The second edition emphasizes patient-specific biomaterials and medical devices, precision medicine approaches, additive manufacturing and digital design, predictive modeling, and clinically driven studies capable of bridging laboratory research and real-world applications. By fostering interdisciplinary collaboration among materials scientists, engineers, biologists, and clinicians, the second edition aims to accelerate the development of tailor-made biomaterials that improve therapeutic outcomes and address the growing demand for individualized dental and orthopedic interventions.

